# Cardiopulmonary bypass management and risk of new-onset atrial fibrillation after cardiac surgery

**DOI:** 10.1093/icvts/ivad153

**Published:** 2023-09-15

**Authors:** Amar Taha, Anders Hjärpe, Andreas Martinsson, Susanne J Nielsen, Mikael Barbu, Aldina Pivodic, Lukas Lannemyr, Lennart Bergfeldt, Anders Jeppsson

**Affiliations:** Department of Molecular and Clinical Medicine, Institute of Medicine, Sahlgrenska Academy, University of Gothenburg, Gothenburg, Sweden; Department of Cardiology, Sahlgrenska University Hospital, Gothenburg, Sweden; Department of Anaesthesia and Intensive Care, Institute of Clinical Sciences, Sahlgrenska Academy, University of Gothenburg, Gothenburg, Sweden; Department of Cardiothoracic Surgery, Sahlgrenska University Hospital, Gothenburg, Sweden; Department of Molecular and Clinical Medicine, Institute of Medicine, Sahlgrenska Academy, University of Gothenburg, Gothenburg, Sweden; Department of Cardiology, Sahlgrenska University Hospital, Gothenburg, Sweden; Department of Molecular and Clinical Medicine, Institute of Medicine, Sahlgrenska Academy, University of Gothenburg, Gothenburg, Sweden; Department of Cardiothoracic Surgery, Sahlgrenska University Hospital, Gothenburg, Sweden; Department of Molecular and Clinical Medicine, Institute of Medicine, Sahlgrenska Academy, University of Gothenburg, Gothenburg, Sweden; Department of Cardiology, Blekinge Hospital, Karlskrona, Sweden; APNC Sweden, Gothenburg, Sweden; Department of Clinical Neuroscience, Institute of Neuroscience and Physiology, Sahlgrenska Academy, University of Gothenburg, Gothenburg, Sweden; Department of Anaesthesia and Intensive Care, Institute of Clinical Sciences, Sahlgrenska Academy, University of Gothenburg, Gothenburg, Sweden; Department of Cardiothoracic Anaesthesiology and Intensive Care, Sahlgrenska University Hospital, Gothenburg, Sweden; Department of Molecular and Clinical Medicine, Institute of Medicine, Sahlgrenska Academy, University of Gothenburg, Gothenburg, Sweden; Department of Cardiology, Sahlgrenska University Hospital, Gothenburg, Sweden; Department of Molecular and Clinical Medicine, Institute of Medicine, Sahlgrenska Academy, University of Gothenburg, Gothenburg, Sweden; Department of Cardiothoracic Surgery, Sahlgrenska University Hospital, Gothenburg, Sweden

**Keywords:** New-onset postoperative atrial fibrillation, Cardiac surgery, Cardiopulmonary bypass

## Abstract

**OBJECTIVES:**

Cardiopulmonary bypass (CPB) management may potentially play a role in the development of new-onset atrial fibrillation (AF) after cardiac surgery. The aim of this study was to explore this potential association.

**METHODS:**

Patients who underwent coronary artery bypass grafting and/or valvular surgery during 2016–2020 were included in an observational single-centre study. Data collected from the Swedish Web System for Enhancement and Development of Evidence-Based Care in Heart Disease Evaluated According to Recommended Therapies registry and a local CPB database were merged. Associations between individual CPB variables (CPB and aortic clamp times, arterial and central venous pressure, mixed venous oxygen saturation, blood flow index, bladder temperature and haematocrit) and new-onset AF were analysed using multivariable logistic regression models adjusted for patient characteristics, comorbidities and surgical procedure.

**RESULTS:**

Out of 1999 patients, 758 (37.9%) developed new-onset AF. Patients with new-onset postoperative AF were older, had a higher incidence of previous stroke, worse renal function and higher EuroSCORE II and CHA_2_DS_2_-VASc scores and more often underwent valve surgery. Longer CPB time [adjusted odds ratio 1.05 per 10 min (95% confidence interval 1.01–1.08); *P* = 0.008] and higher flow index [adjusted odds ratio 1.21 per 0.2 l/m^2^ (95% confidence interval 1.02–1.42); *P* = 0.026] were associated with an increased risk for new-onset AF, while the other variables were not. A sensitivity analysis only including patients with isolated coronary artery bypass grafting supported the primary analyses.

**CONCLUSIONS:**

CPB management following current guideline recommendations appears to have minor or no influence on the risk of developing new-onset AF after cardiac surgery.

**Figure:**
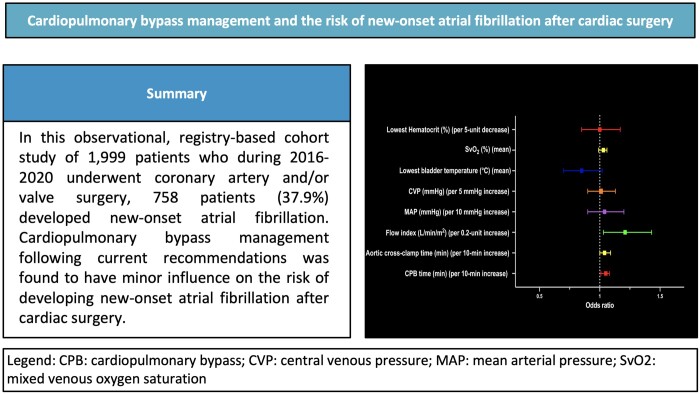

## INTRODUCTION

New-onset postoperative atrial fibrillation (POAF) is a common complication following cardiac surgery. It occurs in 20–60% of cardiac surgery patients depending on the type of procedure [1, 2]. POAF is associated with short- and long-term adverse events, including longer intensive care unit and hospital stay and increased risk for thromboembolic events, heart failure hospitalization and atrial fibrillation (AF) recurrence [3–8]. Despite efforts to prevent POAF, the prevalence has remained unchanged over the years [9].

Cardiopulmonary bypass (CPB) secures sufficient oxygenation and circulation during cardiac surgery. Management in CPB is complex and includes several procedural aspects such as temperature, fluid balance, blood flow, pressure management, anticoagulation and invasive monitoring during cardiac surgery [10, 11]. Several studies have shown that coronary artery bypass grafting (CABG) patients operated with CPB have a higher risk of developing POAF than those operated without CPB, suggesting that CPB may play a role in POAF development [12, 13]. We have not found any previous study investigating the association between POAF and variables involved in CPB management. The aim of this observational study was therefore to explore if potentially modifiable CPB variables are associated with the development of POAF.

## MATERIALS AND METHODS

### Design and population

This is an observational study based on prospectively collected data obtained from 2 registries: the Swedish Cardiac Surgery Registry and the local institutional CPB database. Registry data were merged using the patients’ Swedish personal identification number.

**Figure 1:**
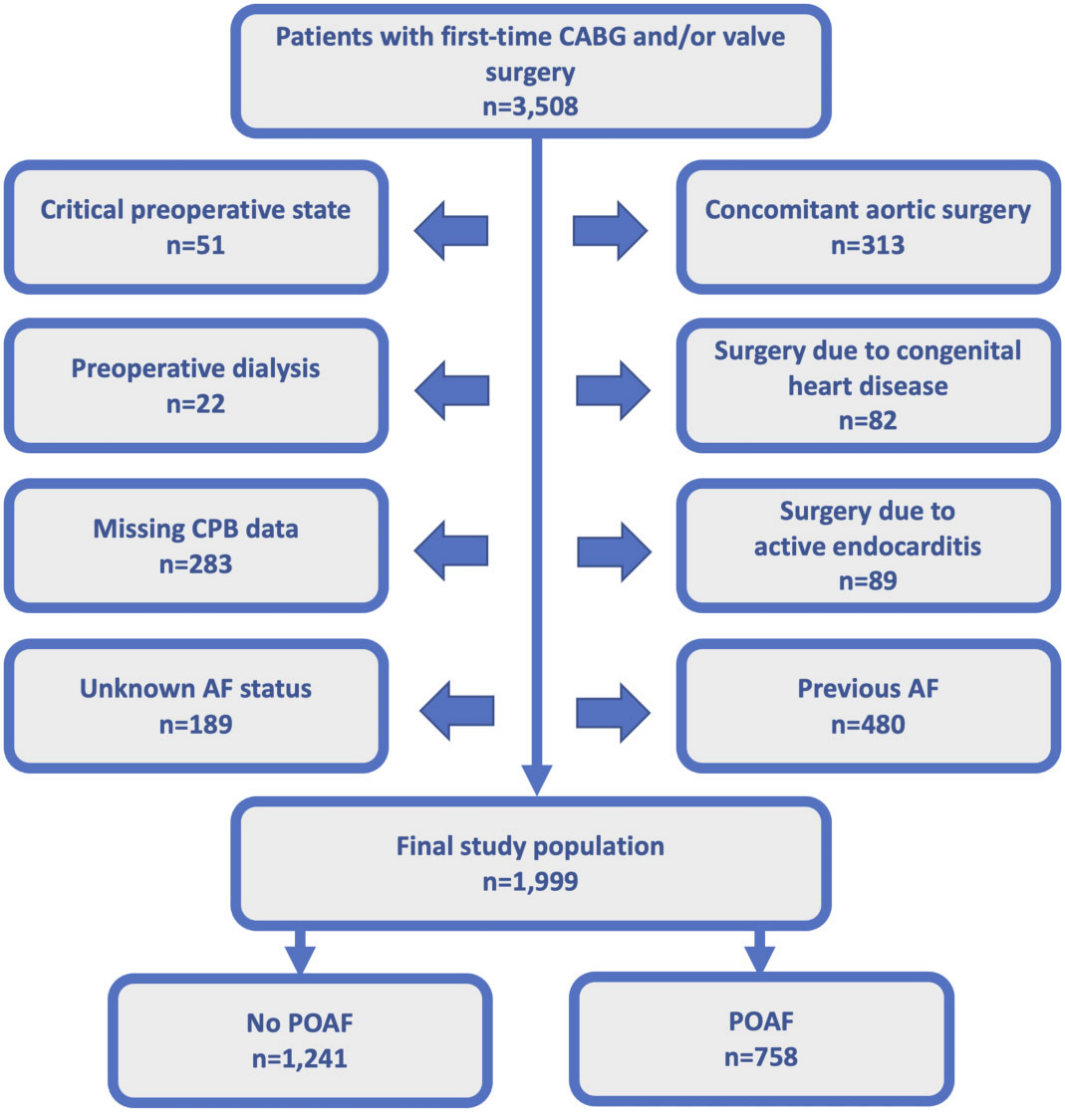
Flow chart of included and excluded patients. AF: atrial fibrillation; CABG: coronary artery bypass grafting; CPB: cardiopulmonary bypass; POAF: new-onset postoperative atrial fibrillation.

A total of 3508 patients who underwent first-time CABG and/or valvular heart surgery at Sahlgrenska University Hospital, Gothenburg, Sweden, between October 2016 and September 2020 were identified. Patients with concomitant aortic surgery, active endocarditis, critical preoperative state, preoperative dialysis, substantially missing CPB data, previous AF or unknown postoperative AF status and those who underwent surgery for congenital heart disease were excluded to achieve a homogeneous study population. The final population therefore comprised 1999 patients (Fig. 1).

This article was written in accordance with the recommendations in the Strengthening the Reporting of Observational Studies in Epidemiology statement [14].

### Data sources

The Swedish Cardiac Surgery Registry is part of the Swedish Web System for Enhancement and Development of Evidence-Based Care in Heart Disease Evaluated According to Recommended Therapies (SWEDEHEART) registry. Since 1992, it has contained clinical and operative information on all patients undergoing cardiac surgery in Sweden, with excellent (>99%) coverage and high validity [15, 16]. All information on patient characteristics, previous comorbidities, laboratory tests, operative data and POAF status was obtained from this registry. Diagnoses were considered present if they were reported to the registry. A detailed list on the variables collected from this registry is provided in Supplementary Material, Table S1.

The local institutional CPB database contains information on several guidelines-recommended [11] physiological monitoring parameters collected during CPB. Using new technology, this information is automatically registered every 20 s during CPB and stored by the LivaNova Connect data management system (LivaNova, Mirandola, Italy). Supplementary Material, Table S2 provides a detailed list on variables registered in and collected from this database.

### Perioperative patient management

Anaesthesia was induced with fentanyl and propofol and maintained with sevoflurane and/or propofol. The CPB circuit was primed with 1100 ml of Ringer’s acetate and 200 ml of mannitol. Non-pulsatile CPB was performed in all patients in accordance with current guidelines (issued by the European Association for Cardio-Thoracic Surgery Council, the Board of Directors of the European Association of Cardiothoracic Anaesthesiology and the Quality and Outcomes Committee of the European Board of Cardiovascular Perfusion) [11]. A mean arterial pressure (MAP) of 50–80 mmHg was maintained with the administration of norepinephrine, phenylephrine or nitroprusside as needed. Steroids were not routinely administered. Cardioprotection was achieved with antegrade and/or retrograde cold blood cardioplegia in all patients, according to the preference of the surgeon.

### New-onset postoperative atrial fibrillation

At our institution, all patients following cardiac surgery and until discharge are monitored by continuous electrocardiography. POAF was considered present if new-onset AF was detected between the time of the surgical procedure and the discharge date and registered in the Swedish Cardiac Surgery Registry as such.

### Statistical analyses

Normally distributed continuous variables are described as mean and standard deviation; non-normally distributed data are given as median and interquartile range. Categorical variables are reported as number and proportion (%). Associations between preoperative and individual CPB variables and POAF were assessed by binary logistic regression. To identify patient characteristics associated with POAF, an age- and sex-adjusted logistic regression model was used, with POAF as the dependent variable. Patient characteristic variables that were significantly associated with POAF [age, body surface area (BSA), previous stroke, operative priority and surgical procedure] were then used for adjustment in the multivariable logistic regression model to study individual CPB variables. In multivariable model 1, CPB variables were adjusted for these patient characteristics. To identify CPB variables independently associated with POAF, a forward and backward stepwise regression model was utilized (model 2), which included all CPB variables significantly associated with POAF in model 1. Restricted natural cubic splines were used to investigate if the relationship between CPB variables and incidence of POAF was linear or non-linear. The analyses showed that analyses based on linear assumptions were sufficient for all CPB variables. Potential collinearity issues were dismissed after reviewing the variance inflation factors for all variables. The results of the logistic regression models are presented as odds ratios (ORs) with 95% confidence intervals (CIs) and *P*-values. A sensitivity analysis, which included only patients who underwent isolated CABG, was performed and adjusted for the same variables as the main analysis. Factors associated with flow index during CPB were explored. For comparison between 3 groups of patients according to flow index tertiles, Mantel–Haenszel Chi-squared trend test was used for dichotomous and ordered categorical variables, Chi-squared test for non-ordered categorical variables and Jonckheere–Terpstra test for continuous variables.

To estimate whether a temporary reduction in flow index or MAP during CPB was associated with POAF development, a flow index of <2.0 l/min/m^2^ or MAP <40 mmHg for ≥1 min during aortic cross-clamp was arbitrarily defined as compromised flow/MAP, treated as a categorical variable and included in the regression models. The SWEDEHEART registry variables had missing data for New York Heart Association class (4.3%) and the CPB database variables had missing data on MAP (2.6%), central venous pressure (CVP) (3.8%), bladder temperature (4.8%) and mixed venous oxygen saturation (SvO_2_) (9.6%). All other variables had missing values in <1% of patients. All tests were two-tailed and conducted at the 0.05 significance level. All analyses were performed using SAS software, version 9.4 (SAS Institute Inc., Cary, NC, USA).

### Ethical statement

The study was conducted according to the Declaration of Helsinki and was approved by the Regional Ethics Committee in Gothenburg (approval No. 2020-06253) who waived the need for individual patient consent.

## RESULTS

### General

A detailed description of patient characteristics and CPB variables in patients with and without POAF is presented in Tables 1 and 2. Out of the 1999 patients included in this study, 458 (22.9%) were women. Patients’ mean age was 67.8 ± 9 years. Isolated CABG was performed in 62.4% of the patients, while 27.9% and 9.7%, respectively, underwent valve surgery and combined CABG and valve surgery. Postoperative AF occurred in 37.9% (*n* = 758) of all patients. The proportion of patients with POAF varied according to the type of surgical procedure. In isolated CABG, POAF occurred in 32.8% of patients, compared with 43.1% of patients receiving valve surgery and 56.2% of those undergoing combined CABG and valve surgery.

**Table 1:** Patient characteristics and comorbidities in patients with and without new-onset postoperative atrial fibrillation

Variable	No POAF, *N* = 1241	POAF, *N* = 758
Female sex	285 (23.0%)	173 (22.8%)
Age (years)	66.1 (9.5)	70.5 (8.3)
BMI (kg/m^2^)	27.5 (4.2)	27.4 (4.4)
BSA (m^2^)	1.98 (0.20)	1.98 (0.20)
Previous PCI	177 (14.3%)	122 (16.1%)
Previous stroke	82 (6.6%)	76 (10.0%)
Diabetes mellitus	317 (25.5%)	185 (24.5%)
Hypertension	882 (71.5%)	554 (73.5%)
Chronic lung disease	68 (5.5%)	48 (6.3%)
Peripheral vascular disease	55 (4.4%)	37 (4.9%)
eGFR (CKD-EPI) (ml/min)	78.6 (17.4)	73.7 (18.2)
Haemoglobin before surgery (g/l)	139.4 (14.1)	137.6 (13.9)
LVEF		
>50%	959 (77.4%)	576 (76.2%)
31–50%	229 (18.5%)	147 (19.4%)
≤30%	51 (4.1%)	33 (4.4%)
NYHA functional class		
I	341 (28.8%)	177 (24.2%)
II	537 (45.4%)	336 (46.0%)
III	265 (22.4%)	196 (26.8%)
IV	41 (3.5%)	21 (2.9%)
Operative priority		
Elective	641 (51.7%)	453 (59.8%)
Urgent	548 (44.2%)	274 (36.1%)
Emergency	51 (4.1%)	31 (4.1%)
Surgical procedure		
Isolated CABG	839 (67.6%)	409 (54.0%)
Isolated valve	317 (25.5%)	240 (31.7%)
CABG + valve	85 (6.8%)	109 (14.4%)
EuroSCORE II (%)		
Mean (SD)	1.8 (1.8)	2.26 (2.25)
Median (IQR)	1.3 (0.9–2.0)	1.6 (1.0–2.7)
CHA_2_DS_2_-VASc score		
Mean (SD)	3.2 (1.6)	3.7 (1.5)
Median (IQR)	3 (2–4)	4 (3–5)

Data are presented as mean (SD), median (IQR) or number (%).

BMI: body mass index; BSA: body surface area; CABG: coronary artery bypass grafting; CHA_2_DS_2_-VASc: congestive heart failure, hypertension, age ≥75 years, diabetes mellitus, previous stroke or TIA, vascular disease, age 65–74 years, sex category female; CKD-EPI: Chronic Kidney Disease Epidemiology Collaboration; eGFR: estimated glomerular filtration rate; IQR: interquartile range; LVEF: left ventricular ejection fraction; NYHA: New York Heart Association; PCI: percutaneous coronary intervention; POAF: new-onset postoperative atrial fibrillation; SD: standard deviation.

**Table 2:** Cardiopulmonary bypass variables in patients with and without new-onset postoperative atrial fibrillation

Variable	No POAF, *N* = 1241	POAF, *N* = 758
CPB time (min)		
Mean (SD)	79 (30)	85 (36)
Median (IQR)	73 (61–90)	78 (63–95)
Aortic cross-clamp time (min)		
Mean (SD)	55 (25)	61 (29)
Median (IQR)	51 (40–66)	55 (42–70)
Flow index during bypass (l/min/m^2^)		
Mean (SD)	2.45 (0.12)	2.47 (0.11)
Median (IQR)	2.5 (2.4–2.5)	2.5 (2.4–2.5)
Compromised flow index during bypass	266 (21.4%)	145 (19.1%)
MAP during bypass (mmHg)		
Mean (SD)	58 (8)	59 (0)
Median (IQR)	59 (54–63)	59 (54–63)
Compromised MAP during bypass	682 (55.5%)	429 (57.1%)
CVP during bypass (mmHg)		
Mean (SD)	4.7 (4.2)	4.5 (4.3)
Median (IQR)	4.5 (2.1–7.0)	4.3 (2.2–6.5)
Lowest bladder temperature during bypass (°C)		
Mean (SD)	35.7 (0.5)	35.7 (0.6)
Median (IQR)	36 (31–37)	36 (32–37)
SvO_2_ during bypass (%)		
Mean (SD)	76.6 (3.1)	77.0 (3.2)
Median (IQR)	77 (75–79)	77 (75–79)
Lowest haematocrit during bypass (%)		
Mean (SD)	28.3 (3.6)	28.0 (3.5)
Median (IQR)	29 (26–31)	28 (26–31)

Data are presented as mean (SD), median (IQR) or number (%).

CPB: cardiopulmonary bypass; CVP: central venous pressure; IQR: Interquartile range; MAP: mean arterial pressure; POAF: new-onset postoperative atrial fibrillation; SD: standard deviation; SvO_2_: mixed venous oxygen saturation.

Patients with POAF were generally older and more often had a previous stroke, lower estimated glomerular filtration rate (eGFR) and higher EuroSCORE II and CHA_2_DS_2_-VASc scores (Table 1). Postoperative AF occurred more often when the cardiac surgical procedure was elective than when an emergency surgery was performed.

### Patient characteristics associated with postoperative atrial fibrillation

The age- and sex-adjusted analysis investigating the association between preoperative patient characteristics and POAF showed that advanced age (OR 1.77 per 10-year increase [95% CI 1.58–1.98]), large body surface (OR 1.36 per 0.5-m^2^ increase [1.03–1.81]), previous stroke (OR 1.39 [0.99–1.94]), valve surgery (OR 1.69 [1.36–2.10]), combined CABG and valvular surgery (OR 2.05 [1.49–2.81]) and elective priority (OR for urgent operative priority 0.73 [0.60–0.89]) were associated with an increased risk of developing POAF (Supplementary Material, Table S3). These variables were used for adjustment in the multivariable logistic regression models.

### Cardiopulmonary bypass variables associated with postoperative atrial fibrillation

CPB variables in patients with and without POAF are presented in Table 2. Results of the univariate and multivariable logistic regression models are presented in Table 3. In the unadjusted univariate analysis, CPB and aortic cross-clamp times, flow index, SvO_2_ and lowest haematocrit during bypass were significantly associated with POAF occurrence. Model 1, adjusted for age, BSA, previous stroke, operative priority and type of surgical procedure, showed that CPB time [adjusted OR (aOR) 1.05 per 10-min increase [95% CI 1.01–1.08]; *P* = 0.007], aortic cross-clamp time (aOR 1.04 per 10-minute increase [1.00–1.09]; *P* = 0.05) and flow index (aOR 1.21 per 0.2-l/min/m^2^ increase [1.03–1.43]; *P* = 0.023) were associated with POAF. There were no significant associations between MAP, CVP, SvO_2_, lowest haematocrit during CPB and compromised flow or MAP, on the one hand, and POAF, on the other hand, in this model. The stepwise regression analysis (model 2) showed that CPB time (aOR 1.05 per 10-minute increase [1.01–1.08]; *P* = 0.008) and flow index during bypass (aOR 1.21 per 0.2-unit increase [1.02–1.42]; *P* = 0.026) remained the only CPB factors independently associated with POAF development.

**Table 3:** Unadjusted and adjusted (model 1) logistic regression and stepwise (forward and backward) regression (model 2) models for new-onset postoperative atrial fibrillation using cardiopulmonary bypass variables as main effect variables

Variable	Unadjusted			Model 1			Model 2	
	OR (95% CI)	*P*-Value	AUC	OR (95% CI)	*P*-Value	AUC	OR (95% CI)	*P*-Value
CPB time (min) (per 10 min increase)	1.06 (1.03–1.10)	<0.0001	0.55	1.05 (1.01–1.08)	0.007	0.67	1.05 (1.01–1.08)	0.008
Aortic cross-clamp time (min) (per 10 min increase)	1.09 (1.05–1.13)	<0.0001	0.56	1.04 (1.00–1.09)	0.050	0.66		
Flow index during bypass (mean) (per 0.2 units increase)	1.24 (1.07–1.45)	0.005	0.54	1.21 (1.03–1.43)	0.023	0.66	1.21 (1.02–1.42)	0.026
Compromised flow index during bypass (ref no)	0.87 (0.69–1.09)	0.22	0.51	0.82 (0.65–1.04)	0.100	0.66		
MAP during bypass (mean) (per 10 units increase)	1.04 (0.91–1.19)	0.54	0.51	1.04 (0.91–1.20)	0.56	0.66		
Compromised MAP during bypass (ref no)	1.07 (0.89–1.28)	0.48	0.51	1.09 (0.90–1.32)	0.38	0.66		
CVP during bypass (mean) (per 5 units increase)	0.96 (0.86–1.07)	0.44	0.51	1.01 (0.90–1.13)	0.88	0.66		
Lowest bladder temperature during bypass (°C)	0.86 (0.73–1.02)	0.080	0.52	0.85 (0.70–1.02)	0.090	0.67		
SvO_2_ (%) during bypass (mean)	1.04 (1.01–1.07)	0.007	0.54	1.03 (0.99–1.06)	0.120	0.66		
Lowest haematocrit (%) during bypass (per 5 units decrease)	1.15 (1.01–1.30)	0.031	0.53	1.00 (0.85–1.17)	0.97	0.66		

Model 1: adjusted for age, sex, BSA, previous stroke, operative priority and surgical procedure. Model 2: stepwise (forward and backward) regression adjusted for age, sex, BSA, previous stroke, operative priority and surgical procedure. AUC for this model is 0.67.

AUC: area under the curve; BSA: body surface area; CI: confidence interval; CPB: cardiopulmonary bypass; CVP: central venous pressure; MAP: mean arterial pressure; OR: odds ratio; SvO_2_: mixed venous oxygen saturation.

The sensitivity analysis included 1248 patients who underwent isolated CABG. Out of these, 409 patients (32.8%) developed POAF. The patients are described in Supplementary Material, Table S4, and the logistic regression models are presented in Supplementary Material, Table S5. The results of the sensitivity analysis support the main analysis.

In an additional analysis, factors associated with flow index during bypass were explored. This analysis showed lower body mass index and BSA, history of previous PCI, diabetes, hypertension, lower eGFR and haemoglobin were associated with a higher flow index during bypass (Supplementary Material, Table S6).

## DISCUSSION

In this observational study of 1999 patients undergoing CABG and/or valve surgery, we explored the associations between CPB variables and the risk for POAF. Longer CPB time and higher flow index were associated with an increased risk for POAF, while MAP, CVP, SvO_2_, body temperature and haematocrit had no significant association with risk for developing POAF.

Several potential mechanisms for POAF development have been proposed, including systemic and local inflammation, oxidative stress and electrolyte imbalance induced by the surgical procedure performed in vulnerable patients [1, 17]. However, no exact mechanism has yet been identified. And despite refinements in surgical procedures, anaesthesia, perfusion and postoperative care, the incidence of POAF has, over the years, remained unchanged [9].

The role of CPB in the development of POAF, if any, is not clarified. CPB has evolved into a standardized procedure for which international professional associations have published evidence-based guidelines with explicit recommendations to ensure adequate perfusion and minimize organ injury during surgery [11]. However, CPB triggers an acute inflammatory response through a complex process that involves several mechanisms including contact between the blood products and the artificial surfaces of extracorporeal circulation, surgical trauma and ischaemia–reperfusion trauma [18]. All these mechanisms, working in synergy, provoke a systemic inflammatory process that might contribute to the occurrence of several postoperative complications including POAF. Notably, CABG without CPB is associated with a lower incidence of POAF in comparison with CABG with CPB [12, 13]. Previous studies investigating pre- and perioperative predictors of POAF have described long CPB time as an independent risk factor for POAF. However, no other CPB parameters have previously been explored [19, 20].

In the present study, longer CPB time was independently associated with the development of POAF, which is in line with previous studies [19, 20]. Indeed, more complex cardiac surgical procedures are associated with longer CPB times, as are procedures with complications. However, it may be beneficial, even from a POAF perspective, to keep the CPB time as short as possible, without jeopardizing the patient’s safety and/or surgical quality. Furthermore, the high incidence of POAF observed following more complex surgical procedures, such as combined CABG and valve or multiple valve surgery [1], may in addition to the larger surgical trauma also be due to the longer CPB duration.

Some CPB variables, such as temperature and blood flow, are directly modifiable during surgery, while others such as MAP, SvO_2_ and haematocrit can be modified by a combination of blood flow alterations, ventilation adjustments, infusions of fluids and blood products and vasoactive drugs. It is noteworthy that changes in MAP, CVP, SvO_2_, body temperature and low haematocrit were, in the present study, not significantly associated with risk for POAF. This may be explained, at least in part, by the standardized CPB management with limited individual variations between patients. The optimal MAP during CPB is still debated and rather than targeting an arbitrary MAP, an individualized MAP target based on the patient’s auto-regulatory range has been suggested [10]. The mean MAP in the present study was almost identical in patients with and patients without POAF (59.0 vs 58.8 mmHg), suggesting that an MAP within current recommendations does not influence POAF risk. Notably, compromised MAP (arbitrarily defined as an MAP of <40 mmHg for ≥1 min during aortic cross-clamp) was still not associated with POAF.

One determinant of MAP is blood flow. During CPB, the target flow is determined based on BSA and body temperature and is, in accordance with current CPB guidelines, usually set between 2.2 and 2.8 l/min/m^2^ [11]. The mean blood flow in the present study was 2.42 l/min/m^2^, with small individual variations (standard deviation 0.12 l/min/m^2^). Increased flow index during CPB was independently associated with the risk of POAF occurrence, although the absolute difference in blood flow index between POAF and non-POAF patients was marginal (0.02 l/min/m^2^). Factors associated with a higher flow index in this study included diabetes, hypertension, lower eGFR and a higher CHA_2_DS_2_-VASc score indicating a higher burden of comorbidities, which subsequently is associated with a higher risk for POAF [1]. Low GFR may also in itself explain the higher flow index in POAF patients, since it is common to have higher blood flow in these patients to ensure adequate renal oxygen delivery [11, 21]. Additionally, it may be speculated that an increased flow might be accompanied by electrolyte disturbances, which might also lead to developing POAF. Furthermore, low haematocrit during CPB is associated with several complications after cardiac surgery, including stroke, myocardial infarction, renal and multi-organ failure and mortality [22, 23]. However, low haematocrit was not associated with POAF in the present study.

Finally, we found in this study that POAF occurrence was more common in patients undergoing elective surgical procedures. In this study, 64.5%% of the elective procedures, 16.8% of the urgent and 7.3% of the emergent procedures involved valvular surgery (Supplementary Material, Table S7). As the risk for POAF is higher following valve and combined cardiac surgery than after isolated CABG [8], the type of the surgical procedure is the most likely explanation for the association between POAF and operative priority.

### Limitations

Our present study has both limitations and strengths. The limitations include potential confounding due to the lack of information on postoperative conditions, such as electrolyte imbalance, ventilator and inotrope use and postoperative infections, as well as lack of detailed information about echocardiographic variables other than left ventricular ejection fraction, which could potentially contribute to the risk of POAF development. In addition, we had no information on arterial blood gas analyses during CPB as these variables were not automatically collected. Therefore, the measurement of oxygen delivery (DO_2_) was not available for analysis. Finally, information about POAF episodes occurring after hospital discharge, perioperative medications and the type of valvular surgery is lacking.

However, several preoperative variables, including all those recommended by the Society of Cardiovascular Anesthesiologists/European Association of Cardiothoracic Anaesthetists for POAF prediction [24], were included in the analyses. Other strengths include a large study population, automatic registration of CPB variables every 20 s, electronic transfer of the variables to the dataset and use of the SWEDEHEART registry.

## CONCLUSION

Longer CPB time and higher flow index were in this study independently associated with an increased risk for POAF. Changes in MAP, CVP, SvO_2_, body temperature and haematocrit and comprised flow or MAP showed no significant association with the risk of POAF development. Taken together, the results suggest that CPB management based on current recommendations appears to have a minor influence on the risk of developing POAF after cardiac surgery.

## Supplementary Material

ivad153_Supplementary_Data

## Data Availability

The data underlying this article will be available on reasonable request to the corresponding author, with the permission of SWEDEHEART.

## ABBREVIATIONS

AF: Atrial fibrillation
aOR: Adjusted odds ratio
BSA: Body surface area
CABG: Coronary artery bypass grafting
CHA_2_DS_2_-VASc: Congestive heart failure, hypertension, age ≥75 years, diabetes mellitus, previous stroke or TIA, vascular disease, age 65–74 years, sex category female
CI: Confidence interval
CPB: Cardiopulmonary bypass
CVP: Central venous pressure
eGFR: Estimated glomerular filtration rate
MAP: Mean arterial pressure
OR: Odds ratio
POAF: New-onset postoperative atrial fibrillation
SvO_2_: Mixed venous oxygen saturation
