# Supplementary material for: Cardiopulmonary bypass management and risk of new-onset atrial fibrillation after cardiac surgery
Source: Interdiscip Cardiovasc Thorac Surg. 2023 Sep 15;37(3):ivad153. doi: 10.1093/icvts/ivad153 (PMC10533753; doi:10.1093/icvts/ivad153)
Supplement: ivad153_Supplementary_Data [file ivad153_supplementary_data.docx]

**SUPPLEMENTARY MATERIAL**

**Table S1: Variables collected from the Swedish Cardiac Surgery Register.**

| Sex |
| --- |
| Age |
| Body mass index (BMI) (kg/m^2^) |
| Body surface area (BSA) (m^2^) |
| Previous percutaneous coronary intervention (PCI) |
| History of stroke |
| History of diabetes mellitus |
| History of hypertension |
| History of chronic lung disease |
| History of atrial fibrillation (AF) |
| History of peripheral arterial disease |
| Estimated glomerular filtration rate (mL/min) (eGFR) |
| Preoperative haemoglobin (g/L) |
| Left ventricular ejection fraction (LVEF) |
| New York Heart Association (NYHA) functional class |
| Operative priority |
| Type of cardiac surgical procedure |
| EuroSCORE II |
| CHA_2_DS_2_-VASc score |
| New-onset postoperative atrial fibrillation (POAF) |

**Table S2: Variables collected from the institutional cardiopulmonary bypass database.**

| Cardiopulmonary bypass (CPB) time (min) |
| --- |
| Aortic cross-clamp time (min) |
| Mean flow index during bypass (L/min/m^2^) |
| Compromised flow index during bypass (Yes/No) |
| Mean arterial blood pressure (MAP) during bypass (mmHg) |
| Compromised MAP during bypass (Yes/No) |
| Central venous pressure (CVP) during bypass (mmHg) |
| Bladder temperature during bypass (℃) |
| Mixed venous oxygen saturation (SvO_2_ %) |
| Haematocrit during bypass (%) |

**Table S3: Logistic regression for patient characteristics, adjusted for age and sex, for development of new-onset postoperative atrial fibrillation.**

| **Variable** | **OR (95% CI)** | **p-value** |
| --- | --- | --- |
| Sex (ref female) | 1.11 (0.89–1.38) | 0.37 |
| Age (per 10-year increase) | 1.77 (1.58–1.98) | <0.001 |
| BMI (per 5-kg/m^2^ increase) | 1.08 (0.96–1.20) | 0.20 |
| BSA (per 0.5-unit increase) | 1.36 (1.03–1.81) | 0.032 |
| Previous PCI (ref No.) | 1.15 (0.89–1.48) | 0.30 |
| Previous stroke (ref No.) | 1.39 (0.99–1.94) | 0.050 |
| Diabetes mellitus (ref No.) | 0.93 (0.75–1.15) | 0.49 |
| Hypertension (ref No.) | 0.87 (0.70–1.08) | 0.21 |
| Chronic lung disease (ref No.) | 1.06 (0.72–1.57) | 0.76 |
| Peripheral vascular disease (ref No.) | 0.91 (0.59–1.41) | 0.68 |
| eGFR (CKD-EPI, per 10-unit increase) | 0.96 (0.90–1.01) | 0.13 |
| eGFR (CKD-EPI, cat.) (ref 60–<90) |  |  |
| <30 | 2.13 (0.93–4.86) | 0.070 |
| 30–<45 | 1.30 (0.82–2.05) | 0.26 |
| 45–<60 | 1.09 (0.82–1.46) | 0.54 |
| ≥90 | 1.05 (0.82–1.35) | 0.69 |
| Haemoglobin before surgery, g/L (per 10-unit increase) | 0.98 (0.91–1.05) | 0.58 |
| Haemoglobin before surgery, g/L (cat.) † (ref normal) |  |  |
| Low | 0.97 (0.76–1.23) | 0.78 |
| High | 0.69 (0.27–1.81) | 0.45 |
| LVEF (ref >50%) |  |  |
| 31–50% | 1.03 (0.81–1.31) | 0.79 |
| ≤30% | 1.12 (0.70–1.77) | 0.65 |
| NYHA functional class (ref I) |  |  |
| II | 1.10 (0.87–1.39) | 0.41 |
| III | 1.27 (0.97–1.66) | 0.080 |
| IV | 0.96 (0.54–1.70) | 0.88 |
| Operative priority (ref elective) |  |  |
| Urgent | 0.73 (0.60–0.89) | 0.002 |
| Emergency | 0.90 (0.56–1.45) | 0.66 |
| Surgical procedure (ref CABG) |  |  |
| Valve | 1.69 (1.36–2.10) | <0.001 |
| CABG+valve | 2.05 (1.49–2.81) | <0.001 |
| EuroSCORE II (mortality %) | 1.04 (0.99–1.10) | 0.090 |
| CHA_2_DS_2_-VASc score | 1.01 (0.93–1.09) | 0.79 |
| †Low haemoglobin (Hb) (g/L): women <120, men <130; normal Hb: women 120–155, men 130–170; high Hb: women >155, men >170.  BMI: body mass index; BSA: body surface area; CABG: coronary artery bypass grafting; CHA_2_DS_2_-VASc: Congestive heart failure, Hypertension, Age≥75 years, Diabetes mellitus, previous Stroke or TIA, Vascular disease, Age 65-74 years, Sex category female; CKD-EPI: Chronic Kidney Disease Epidemiology Collaboration; eGFR: estimated glomerular filtration rate; LVEF: left ventricular ejection fraction; NYHA: New York Heart Association; PCI: percutaneous coronary intervention; POAF: new-onset postoperative atrial fibrillation. | | |

**Table S4: Patients characteristics, comorbidities and cardiopulmonary bypass variables in patients with and without new-onset postoperative atrial fibrillation following isolated CABG.**

| **Variable** | | **No POAF N=839** | **POAF N=409** |
| --- | --- | --- | --- |
| ***Patient characteristics and comorbidities*** | |  |  |
| Female sex | | 154 (18.4%) | 64 (15.6%) |
| Age (years) | | 66.1 (8.8) | 70.4 (7.4) |
| BMI (kg/m^2^) | | 27.7 (4.0) | 27.8 (4.3) |
| BSA (m^2^) | | 1.99 (0.19) | 2.00 (0.19) |
| Previous PCI | | 149 (17.8%) | 94 (23.0%) |
| Previous stroke | | 59 (7.0%) | 41 (10.0%) |
| Diabetes | | 253 (30.2%) | 121 (29.7%) |
| Hypertension | | 656 (78.7%) | 320 (78.6%) |
| Chronic lung disease | | 48 (5.7%) | 23 (5.7%) |
| Peripheral vascular disease | | 42 (5.0%) | 26 (6.4%) |
| eGFR (CKD-EPI) | | 79.1 (16.9) | 74.1 (18.7) |
| Haemoglobin before surgery (g/l) | | 139.6 (13.8) | 138.3 (14.2) |
| LVEF | |  |  |
| >50% | | 630 (75.3%) | 295 (72.1%) |
| 31%-50% | | 170 (20.3%) | 94 (23.0%) |
| ≤30% | | 37 (4.4%) | 20 (4.9%) |
| NYHA functional class | |  |  |
| I | | 315 (40.1%) | 148 (38.2%) |
| II | | 343 (43.6%) | 164 (42.4%) |
| III | | 103 (13.1%) | 68 (17.6%) |
| IV | | 25 (3.2%) | 7 (1.8%) |
| Operative priority | |  |  |
| Elective | | 297 (35.4%) | 155 (37.9%) |
| Urgent | | 492 (58.7%) | 229 (56.0%) |
| Emergency | | 49 (5.8%) | 25 (6.1%) |
| Euroscore II (mortality %) | | 1.73 (1.57) | 2.15 (2.04) |
| CHA_2_DS_2_-VASc score | | 3.3 (1.6) | 3.7 (1.5) |
| ***Heart-lung machine variables*** | |  |  |
| CPB time (min)  Mean (SD)  Median (IQR) | | 72.3 (21.4) 68 (58–84) | 74.8 (26.2) 72 (59–86) |
| Aortic x-clamp time (min)  Mean (SD)  Median (IQR) | | 47.2 (15.2) 45 (37–55) | 48.1 (16.6) 46 (38–57) |
| Flow index during bypass (L/min/m2)  Mean (SD)  Median (IQR) | | 2.44 (0.13) 2.4 (2.4–2.5) | 2.45 (0.11) 2.5 (2.4–2.5) |
| Compromised flow index during bypass | | 196 (23.4%) | 88 (21.5%) |
| MAP during bypass (mmHg)  Mean (SD)  Median (IQR) | | 58.6 (6.7) 59 (54–63) | 58.9 (6.9) 59 (54–63) |
| Compromised MAP during bypass | | 434 (51.8%) | 213 (52.5%) |
| CVP during bypass (mean)  Mean (SD)  Median (IQR) | | 5.15 (4.07) 4.9 (2.7–7.4) | 5.09 (3.91) 4.7 (2.8–7.3) |
| Lowest bladder temperature during bypass (℃) | | 35.7±0.5 36 (34 - 37) | 35.6±0.6 36 (32 - 37) |
| SvO2 during bypass (%)  Mean (SD)  Median (IQR) | | 76.5 (3.2) 77 (75–79) | 76.9 (3.2) 77 (75–79) |
| Lowest Haematocrit during bypass  Mean (SD)  Median (IQR) | | 28.4 (3.5) 29 (26–31) | 28.3 (3.4) 29 (26–31) |
| Data are presented as mean (SD), median (IQR) or number (%). BMI: body mass index; BSA: body surface area; CABG: coronary artery bypass grafting; CPB: cardiopulmonary bypass; CHA2DS2-VASc: Congestive heart failure, Hypertension, Age≥75 years, Diabetes mellitus, previous Stroke or TIA, Vascular disease, Age 65-74 years, Sex category female; CKD-EPI: Chronic Kidney Disease Epidemiology Collaboration; CVP: central venous pressure; eGFR: estimated glomerular filtration rate; IQR: Interquartile range; LVEF: left ventricular ejection fraction; NYHA: New York Heart Association; PCI: percutaneous coronary intervention; POAF: new-onset postoperative atrial fibrillation; SD: standard deviation; SvO2: mixed venous oxygen saturation. | | |  |

**Table S5: Logistic regression fully adjusted for age, sex, body surface area, previous stroke, operative priority, and surgical procedure in patients with isolated CABG for new-onset postoperative atrial fibrillation using cardiopulmonary bypass variables as main effect variables.**

| **Variable** | **OR (95% CI)** | **p-value** | **AUC** |
| --- | --- | --- | --- |
| CPB time (min) (per 10 min increase) | 1.05 (1.00 - 1.11) | 0.07 | 0.65 |
| Aortic x-clamp time (min) (per 10 min increase) | 1.04 (0.96 - 1.12) | 0.38 | 0.65 |
| Flow index during bypass, L/min/m^2^ (mean) (per 0.2-unit increase) | 1.26 (1.02 - 1.56) | 0.029 | 0.65 |
| Compromised flow index during bypass | 0.86 (0.64 - 1.15) | 0.30 | 0.65 |
| MAP during bypass (mean) (per 10-unit increase) | 1.12 (0.93 - 1.35) | 0.22 | 0.65 |
| Compromised MAP during bypass | 1.13 (0.88 - 1.44) | 0.35 | 0.65 |
| CVP during bypass (mean) (per 5-unit increase) | 1.02 (0.87 - 1.19) | 0.83 | 0.65 |
| Lowest bladder temperature during bypass, ℃ (per ℃) | 0.74 (0.58 - 0.96) | 0.021 | 0.66 |
| SvO2 during bypass % (mean) | 1.01 (0.97 - 1.06) | 0.53 | 0.65 |
| Lowest haematocrit during bypass, % (per 5-unit decrease) | 0.96 (0.77 - 1.19) | 0.72 | 0.65 |
| AUC: area under the curve; CI: confidence interval; CPB: cardiopulmonary bypass; CVP: central venous pressure; MAP: mean arterial pressure; OR: odds ratio; SvO_2_: mixed venous oxygen saturation. | | | |

**Table S6. Patient characteristics and comorbidities by flow tertiles in patients with and without new-onset postoperative atrial fibrillation.**

|  |  | **Flow index during bypass (mean)** | | |  | |
| --- | --- | --- | --- | --- | --- | --- |
| **Variable** | **Total N=1999** | **Tertile 1 (<2.413) N=659** | **Tertile 2 (2.413-<2.512) N=658** | **Tertile 3 (>=2.512) N=682** | **p-value** | |
| Sex |  |  |  |  | 0.83 | |
| Female | 458 (22.9%) | 158 (24.0%) | 140 (21.3%) | 160 (23.5%) |  | |
| Male | 1541 (77.1%) | 501 (76.0%) | 518 (78.7%) | 522 (76.5%) |  | |
| Age (years)  Mean (SD)  Median (IQR) | 67.8 (9.3) 69 (62–75) | 68.1 (8.6) 69 (63–74) | 67.5 (9.3) 69 (61–74) | 67.8 (10.0) 69 (62–75) | 0.94 | |
| BMI (kg/m2)  Mean (SD)  Median (IQR) | 27.5 (4.2) 27 (25–30) | 28.4 (4.5) 28 (25–31) | 27.5 (4.2) 27 (25–30) | 26.6 (3.9) 26 (24–29) | <0.001 | |
| BSA (m^2^)  Mean (SD)  Median (IQR) | 1.98 (0.20) 2.0 (1.9–2.1) | 2.02 (0.21) 2.0 (1.9–2.2) | 1.98 (0.19) 2.0 (1.8–2.1) | 1.94 (0.18) 1.9 (1.8–2.1) | <0.001 | |
| Previous PCI | 299 (15.0%) | 122 (18.5%) | 94 (14.3%) | 83 (12.2%) | 0.001 | |
| Previous stroke | 158 (7.9%) | 44 (6.7%) | 54 (8.2%) | 60 (8.8%) | 0.15 | |
| Diabetes | 502 (25.1%) | 202 (30.7%) | 163 (24.8%) | 137 (20.1%) | <0.001 | |
| Hypertension | 1436 (72.2%) | 512 (77.9%) | 482 (73.7%) | 442 (65.3%) | <0.001 | |
| Chronic lung disease | 116 (5.8%) | 26 (4.0%) | 50 (7.6%) | 40 (5.9%) | 0.14 | |
| Peripheral vascular disease | 92 (4.6%) | 30 (4.6%) | 26 (4.0%) | 36 (5.3%) | 0.52 | |
| eGFR (Cockroft-Gault) (mL/min)  Mean (SD)  Median (IQR) | 87.8 (30.0) 85 (67–106) | 90.7 (30.8) 88 (70–108) | 88.0 (28.9) 85 (69–106) | 84.8 (29.9) 81 (64–103) | <0.001 | |
| Haemoglobin before surgery (g/L)  Mean (SD)  Median (IQR) | 138.7 (14.0) 139 (130–148) | 141.0 (13.8) 141 (133–150) | 139.6 (13.1) 140 (131–148) | 135.7 (14.7) 137 (127–146) | <0.001 | |
| LVEF |  |  |  |  | 0.58 | |
| Normal (>50%) | 1535 (76.9%) | 501 (76.1%) | 515 (78.3%) | 519 (76.4%) |  | |
| Moderate (31%-50%) | 376 (18.8%) | 125 (19.0%) | 115 (17.5%) | 136 (20.0%) |  | |
| Poor (<=30%) | 84 (4.2%) | 32 (4.9%) | 28 (4.3%) | 24 (3.5%) |  | |
| NYHA |  |  |  |  | 0.044 | |
| I | 518 (27.1%) | 192 (30.6%) | 173 (27.5%) | 153 (23.2%) |  | |
| II | 873 (45.6%) | 259 (41.3%) | 295 (47.0%) | 319 (48.4%) |  | |
| III | 461 (24.1%) | 160 (25.5%) | 139 (22.1%) | 162 (24.6%) |  | |
| IV | 62 (3.2%) | 16 (2.6%) | 21 (3.3%) | 25 (3.8%) |  | |
| Operative priority |  |  |  |  | 0.110 | |
| Elective | 1094 (54.8%) | 340 (51.6%) | 360 (54.8%) | 394 (57.8%) |  | |
| Urgent | 822 (41.1%) | 295 (44.8%) | 271 (41.2%) | 256 (37.5%) |  | |
| Emergency | 82 (4.1%) | 24 (3.6%) | 26 (4.0%) | 32 (4.7%) |  | |
| Surgical procedure |  |  |  |  | <0.001 | |
| CABG | 1248 (62.4%) | 466 (70.7%) | 423 (64.3%) | 359 (52.6%) |  | |
| Valve | 557 (27.9%) | 142 (21.5%) | 179 (27.2%) | 236 (34.6%) |  | |
| CABG+Valve | 194 (9.7%) | 51 (7.7%) | 56 (8.5%) | 87 (12.8%) |  | |
| EuroSCORE II (mortality %)  Mean (SD)  Median (IQR) | 1.97 (1.97) 1.4 (0.9–2.2) | 1.89 (1.73) 1.4 (0.9–2.2) | 1.95 (1.95) 1.4 (0.9–2.2) | 2.05 (2.18) 1.5 (1.0–2.3) | 0.120 | |
| CHA_2_DS_2_-VASc score  Mean (SD)  Median (IQR) | 3.4 (1.6) 3 (2–4) | 3.5 (1.5) 4 (2–5) | 3.3 (1.6) 3 (2–4) | 3.3 (1.6) 3 (2–4) | 0.021 | |
| Data are presented as mean (SD), median (IQR) and number (percentage).  BMI: body mass index; BSA: body surface area; CABG: coronary artery bypass grafting; CHA2DS2-VASc: Congestive heart failure, Hypertension, Age≥75 years, Diabetes mellitus, previous Stroke or TIA, Vascular disease, Age 65-74 years, Sex category female; eGFR: estimated glomerular filtration rate; IQR: interquartile range; LVEF: left ventricular ejection fraction; NYHA: New York Heart Association; PCI: percutaneous coronary intervention; SD: standard deviation | | | | | |  |

**Table S7: Type of surgical procedures according to operative priority.**

|  | Type of Surgical procedure | | |
| --- | --- | --- | --- |
| Operative priority | **Isolated CABG**  **No. (%)** | **Isolated Valve**  **No. (%)** | **CABG+Valve**  **No. (%)** |
| Elective | 539 (35.5) | 786 (51.7) | 195 (12.8) |
| Urgent | 863 (83.2) | 92 (8.9) | 82 (7.9) |
| Emergency | 101 (92.7) | 7 (6.4) | 1 (0.9) |
| CABG: coronary artery bypass grafting | | | |
